# Conductive Alginate–Carbon-Black Bioanode for Immediate Microbial Electrolysis Cell Operation

**DOI:** 10.3390/polym18192428

**Published:** 2026-10-05

**Authors:** Abhishiktha Chiliveru, Vamshi Krishna Bommagani, Avinash Jukanti, Irina Amar Dubrovin, Alex Schechter, Rivka Cahan

**Affiliations:** 1 Department of Chemical Engineering, Ariel University, Ariel 40700, Israel; 2 Department of Chemical Sciences, Ariel University, Ariel 40700, Israel; salex@ariel.ac.il; 3 Research and Development Centre for Renewable Energy, New Technologies, Research Centre (NTC), University of West Bohemia, 30100 Pilsen, Czech Republic

**Keywords:** *Shewanella oneidensis*, Pseudomonas aeruginosa, Carbon-black, carbon-felt anode, alginate matrix, extracellular electron transfer

## Abstract

This study investigated alginate-immobilized planktonic electroactive bacteria directly coated onto a carbon-felt anode as an alternative bioanode configuration without prolonged biofilm development. Carbon-black (CB) nanoparticles were incorporated into the alginate matrix to enhance conductivity and facilitate extracellular electron transfer (EET). The electrochemical performance of alginate-immobilized *Shewanella oneidensis* and *Pseudomonas aeruginosa* was systematically evaluated by examining the effects of bacterial density and CB-nanoparticle loading. For both bacterial systems, the optimal bacterial density was OD_600_ 0.5. At this bacterial density, *S. oneidensis* and *P. aeruginosa* achieved current densities of 4.08 ± 0.18 and 1.93 ± 0.18 A/m^2^, respectively. The incorporation of CB-nanoparticles significantly enhanced electrochemical performance with an optimal loading of 3 mg. Under these conditions, the current densities increased to 5.24 ± 0.31 A/m^2^ for *S. oneidensis* and 3.34 ± 0.25 A/m^2^ for *P. aeruginosa*, accompanied by a reduction in charge transfer resistance. SEM analysis confirmed the successful incorporation of bacterial cells and CB-nanoparticles within the alginate matrix. The enhanced electrochemical performance was attributed to conductive pathways formed by CB-nanoparticles, which facilitated electron transfer between the immobilized bacterial cells and the anode. Overall, these findings demonstrated the potential of conductive alginate+carbon-black anodes to provide effective strategies for improving EET.

## 1. Introduction 

Bioelectrochemical systems, particularly microbial electrolysis cells (MECs), have emerged as a promising technology for sustainable energy generation and wastewater treatment using exoelectrogenic bacteria [1]. These bacteria break down organic material for ATP production, which is followed by electron and proton generation. Exoelectrogenic bacteria can be utilized in MEC_S_ to generate current by transporting these electrons outside the cell toward the anode. The electrons as well as the protons are then transferred to the cathode, where reduction reactions typically lead to the recovery of hydrogen [2].

Traditionally, MEC performance relies on the formation of electroactive biofilms on the anode surface. These facilitate extracellular electron transfer (EET). However, biofilm-based systems have several challenges, such as long startup times and diffusion limitations. To overcome them, alternative strategies have been explored to reduce dependence on biofilm formation. One such approach is to entrap planktonic bacterial cells in a redox-activated gel on the anode surface, thereby enabling highly effective EET and immediate electrochemical activity [3]. However, very few studies have reported on how planktonic electroactive bacteria behave when immobilized, and how well they enhance the electron transport pathways. Among the electroactive bacteria, *Shewanella oneidensis* is a model organism for EET. It utilizes a combination of direct electron transfer via outer-membrane cytochromes and mediated electron transfer (MET) through flavins [4]. In contrast, *Pseudomonas aeruginosa* facilitates MET by secreting redox mediators and phenazines [5]. 

Based on the effective electrochemical communication between microorganisms and electrodes, directly or via mediated systems, cheap and efficient materials were developed with high electrical conductivity, corrosion resistance, and biocompatibility to promote bacterial attachment to the anode and electron transfer [3,6]. The selection of the immobilization matrix plays a critical role. Microbial cell immobilization involves enclosing microorganisms in a controlled environment, providing a protective environment for the cells, and allowing them to operate efficiently [7]. This approach is applied in biotechnology, pharmaceuticals, environmental science, food industries, and biosensors. There are various immobilization methods, including entrapment, gel-based matrices, ion-exchange gelation, and polymer-based matrices. Among these, alginate-gel immobilization is one of the most commonly used methods for biohydrogen production, due to its biocompatibility, mild gelation conditions, cost-effectiveness, simplicity, and ability to form a stable hydrogel in the presence of Ca^2+^. Importantly, alginate provides a hydrated and porous environment that supports cell viability while allowing for the diffusion of substrates, metabolites, and redox mediators. Furthermore, immobilization in alginate ensures uniform cell distribution on the electrode surface and enables the development of a controlled, reproducible bio-interface, which is often difficult to achieve in a conventional biofilm-based system [8,9].

The anode material is a factor that affects the exoelectrogenic bacteria and electrical current generation in MECs. Conventionally, MECs use carbon-based materials such as carbon paper, carbon cloth, carbon fibers, carbon felt, and carbon brushes [10], which are important for microbial attachment and electron transfer. Among these materials, carbon felt as an electrode material offers a large outer and inner surface area, high porosity, biocompatibility, and electrical conductivity. The 3D structure of carbon felt provides active sites for immobilization and promotes efficient microbial adhesion, which helps integrate immobilized planktonic cells [11]. Additionally, carbon nanoparticles, such as carbon black, graphene, and carbon nanotubes, are characterized by their high surface area, porosity, and electrical conductivity, which serve as an effective electron conduit. Carbon black can enhance microbial adhesion and facilitate the retention of redox-active compounds, contributing to improved electrochemical performance. Previous studies have demonstrated the benefits of conductive nanoparticles in enhancing microbial electrochemical performance. Chiliveru et al. reported current densities of 2.52 A/m^2^ and 1.85 A/m^2^ using reduced graphene oxide and activated carbon in systems containing planktonic *G. sulfurreducens* cells [12]. Yu et al. observed a 3.5-fold increase in power density using nitrogen-doped carbon particles (NDCDs) to modify the anode in MFCs inoculated with *S. oneidensis* MR-1 [13]. Similarly, Liu et al. demonstrated that graphene-coated carbon cloth inoculated with *P. aeruginosa* exhibited a 2.7-fold higher power density than an unmodified carbon-cloth anode. These findings highlight the importance of the interaction of microorganisms with carbon nanoparticles in enhancing EET [14]. 

We hypothesized that the synergistic interaction between planktonic electroactive bacteria and conductive carbon-black nanoparticles enables electron transfer via direct cell–electrode contact and mediator-facilitated transport, thereby reducing electron transfer resistance and enhancing system response. 

In this study, planktonic electroactive cells were immobilized within an alginate matrix containing conductive carbon-black nanoparticles, directly coated onto a carbon-felt anode. In this design, alginate serves as the polymeric immobilization matrix, while CB acts as the conductive filler within the composite bioelectrode. The electrochemical performance of *S. oneidensis* and *P. aeruginosa* was investigated to evaluate the influence of distinct electron transfer mechanisms under immobilized conditions. Furthermore, the effects of bacterial density and carbon-black loading were systematically examined to identify how the alginate-based bio-interface influences the bioelectrode’s electrochemical characteristics and extracellular electron transfer. This work provides new insights into the design and optimization of conductive alginate-based bioelectrodes for improving the performance and controllability of microbial electrolysis cells. 

## 2. Materials and Methods

### 2.1. Bacterial Strains and Culture Conditions

Two electroactive bacterial strains, *S. oneidensis* MR-1 and *P. aeruginosa* PAO1, were utilized in this study. *S. oneidensis* was cultured in tryptose soy broth (TSB), while *P. aeruginosa* was grown in Luria–Bertani (LB) broth. Both cultures were incubated at 30 °C with shaking at 100 rpm until the bacterial density reached OD_600_ = 0.2, 0.5, 1.0, 1.5, and 1.7, to evaluate the effect of bacterial density on electrochemical performance.

### 2.2. Anode Preparation and Bacteria Immobilization

**Figure 1:**
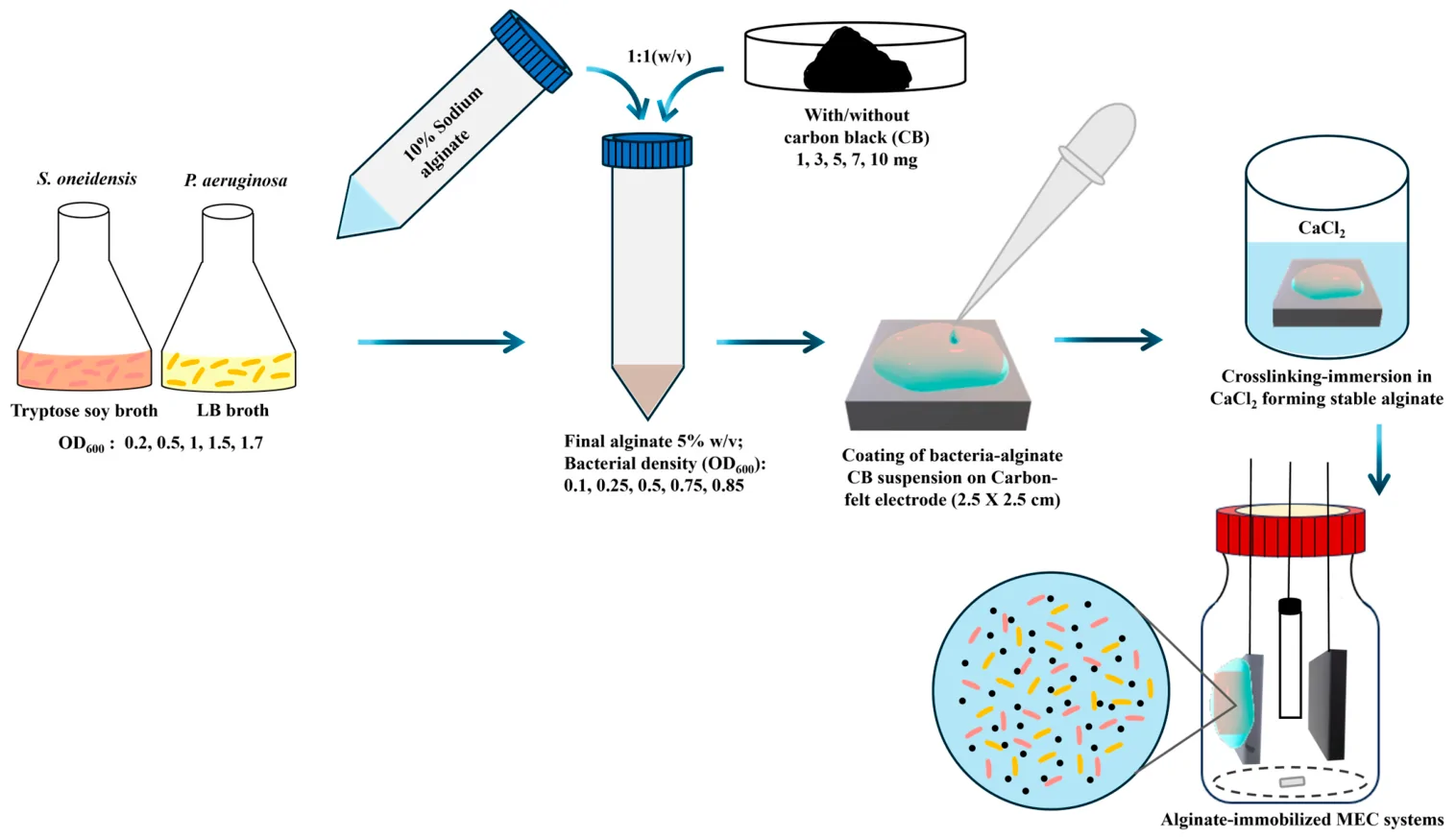
Schematic representation of preparation of alginate-immobilized electroactive bacterial anodes containing CB-nanoparticles for MECs.

A 10% (*w/v*) sodium alginate solution was prepared and used as an immobilization matrix. The planktonic bacterial strains *S. oneidensis* and *P. aeruginosa* at defined OD_600_ values were mixed with the alginate at a 1:1 (*v/v*) ratio to make a homogeneous suspension. Due to this dilution, the final bacterial densities in the immobilization matrix corresponded to OD_600_ 0.1, 0.25, 0.5, 0.75, and 0.85, respectively. The final alginate concentration was 5%. Nanoparticle loadings of 1, 3, 5, 7, and 10 mg/anode were chosen to systematically evaluate the influence of CB loading while avoiding the excessive particle aggregation and blockage that may occur at higher loadings. The effect of CB-nanoparticles as a conductive material was evaluated both in the absence and presence of bacteria. The mixture was uniformly coated onto carbon-felt electrodes (2.5 cm × 2.5 cm). The coated electrodes were crosslinked by immersion in a calcium chloride (CaCl_2_) solution to form a stable alginate matrix, ensuring immobilization of both bacterial cells and carbon-black nanoparticles in the electrode film surface. The prepared electrodes were rinsed gently to remove loosely bound materials and were then used as anodes in the MECs shown in Figure 1. 

### 2.3. MEC Configuration and Operation 

Experiments were carried out in a single-chamber MEC. The alginate+carbon-black and bacteria immobilized on the carbon felt served as the bacterial anode, while the cathode was a carbon cloth coated with 0.5 mg/cm^2^ of 60 wt.% Pt/C (Cloth GDE, Fuel Cells Etc, Bryan, TX, USA). The anode and cathode comprised a geometric area of 6.25 cm^2^. Ag/AgCl served as a reference electrode (1CP, ALS, Tokyo, Japan). Electrodes were connected via titanium wires, which were sealed with epoxy material to ensure electrical insulation. The reactors were filled with 80 mL of lactate medium and 20 mL of 100 mM phosphate buffer. The applied voltage of 0.8 V was supplied by connecting the systems to a MultiEmStat3+ potentiostat (Palmsens, Houten, The Netherlands). Three independent MEC reactors were operated for each experimental condition (n = 3). Data are presented as mean ± standard deviation (SD).

### 2.4. Experimental Design 

The experiments were designed in two sets.

#### 2.4.1. Optimization of Bacterial Density

The influence of bacterial cell density (OD_600_ = 0.1, 0.25, 0.5, 0.75, 0.85) on the current generation was tested.

#### 2.4.2. Optimization of Carbon-Black Loading

Based on the optimal bacterial density obtained from the previous step, carbon-black loadings of 1, 3, 5, 7, and 10 mg/anode were tested to evaluate their synergistic effect on electrochemical performance. Control experiments with alginate-immobilized bacteria without CB-nanoparticles (bacteria only) and alginate-immobilized CB-nanoparticles without bacteria (CB only) were also performed. 

### 2.5. Electrochemical Characterization 

Electrochemical measurements were conducted after preparation of the anode without allowing for prolonged incubation for biofilm development. The alginate matrix enabled direct immobilization of bacterial cells onto the electrode surface. 

The current produced in the MECs was calculated using Ohm’s law. Linear sweep voltammetry (LSV) was performed using a three-electrode configuration, with an alginate-immobilized carbon-felt anode as the working electrode (WE), Ag/AgCl (1CP, ALS, Tokyo, Japan) as the reference electrode (RE), and carbon cloth coated with 0.5 mg/cm^2^ of 60 wt % Pt/C as the counter electrode (CE). The potential was scanned from −0.8 to +0.8 V at a scan rate of 5 mV s^−1^ using a potentiostat (MultiEmStat3+, Palmsens, CL Houten, Vleugelboot, The Netherlands). For comparison among the different experimental conditions, the current density values at 0.8 V were extracted from the LSV curves. Electrochemical impedance spectroscopy (EIS) measurements were conducted to evaluate the resistances associated with the charge transfer (R_ct_). The measurements were performed using a potentiostat (Ivium N-stat; Eindhoven, The Netherlands). All impedance spectra were collected at OCV potential. The resulting EIS spectra were analyzed using ZView software (ZSimWin 3.21). The impedance data were fitted using the equivalent circuit R((Q)(R)(QR)(WR)), where R represents solution resistance, QR elements describe charge transfer, and W represents Warburg impedance associated with diffusion-controlled transport [15] This approach ensures that the observed electrochemical responses primarily reflect the effects of bacterial loading and the loading of the conductive additive, rather than mature biofilm formation. 

### 2.6. Surface and Structural Characterization

The prepared anodes were collected and analyzed by scanning electron microscope (SEM) to understand bacterial and carbon-black attachment on the anode during alginate immobilization. The samples were washed (3×) with PBS, then fixed with 2.5% glutaraldehyde for 1 h. The fixed samples were dehydrated by a series of ethanol–water concentrations (30, 50, 70, 90, 100%) for 10 min each. The dehydrated samples were air-dried and sputtered with gold before SEM observation. These anodes were analyzed using a MAIA3 SEM at ultra-high resolution (TESCAN, Brno, Czech Republic) [12]. 

The anodes were further analyzed by Raman spectroscopy to characterize the Raman spectral features of the bacteria and carbon black, using an XploRA ONE Raman microscope (Horiba Scientific, Kyoto, Japan) with a 532 nm laser.

## 3. Results and Discussion

The electrochemical performance of the alginate-immobilized anode was systematically evaluated using LSV and EIS. Current density values at 0.8 V were extracted from the LSV curves to compare the effects of planktonic bacteria density and the loading of conductive CB-nanoparticles. Four anodes were constructed for MEC systems based on alginate immobilization: alginate and *S. oneidensis*, alginate and *P. aeruginosa*, alginate+*S. oneidensis* combined with CB-nanoparticles, and alginate+*P. aeruginosa* combined with CB-nanoparticles. 

### 3.1. Effect of Bacterial Density on Electrochemical Performance 

**Figure 2:**
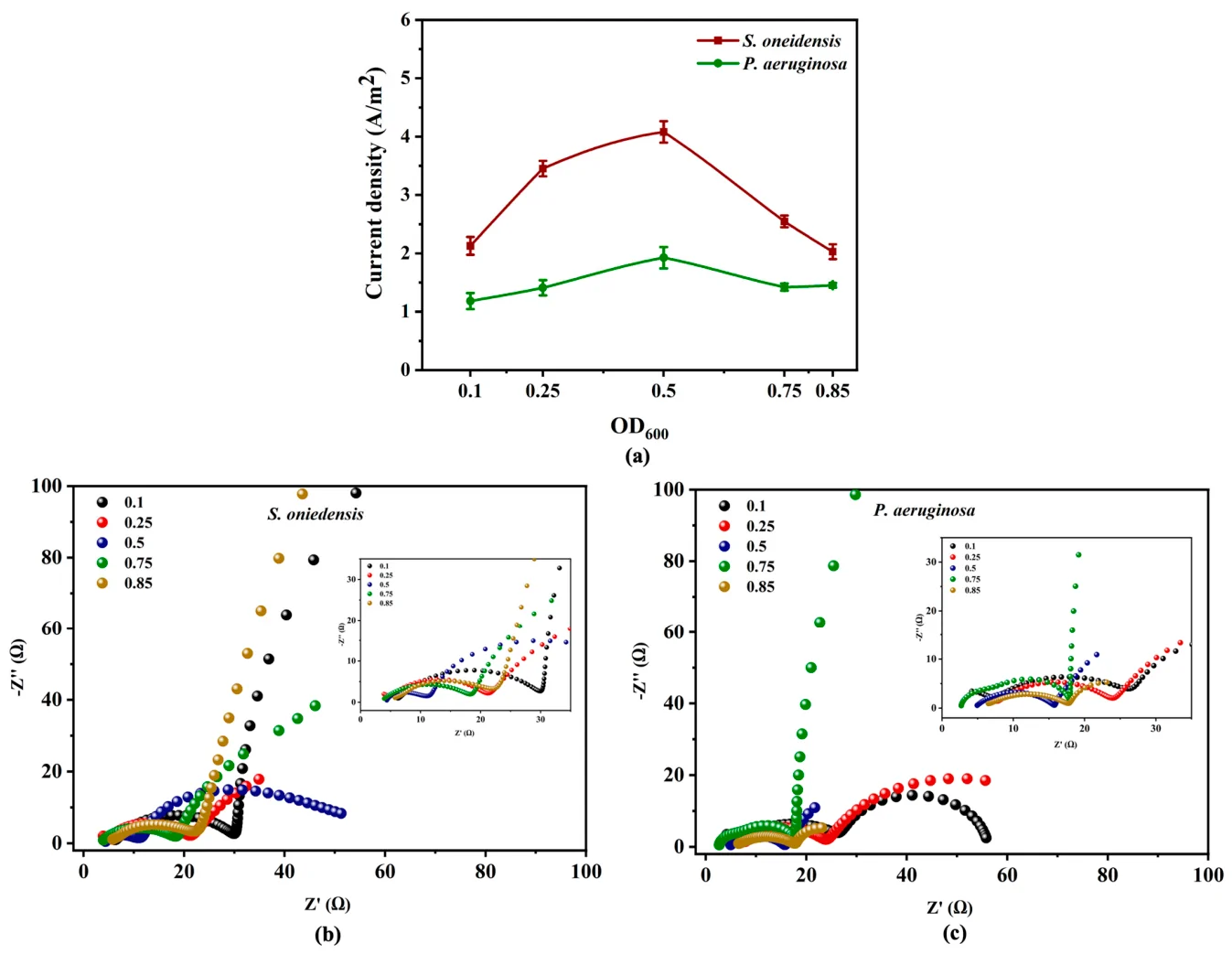
Alginate-immobilized *S. oneidensis* and *P. aeruginosa* MEC systems at different optical densities (ODs): (**a**) current density at 0.8 V extracted from LSV measurements for both bacteria, (**b**) EIS of *S. oneidensis,* and (**c**) EIS of *P. aeruginosa*.

The influence of bacterial density on the current generation was examined by varying the optical density (OD_600_) of the planktonic *S. oneidensis* and *P. aeruginosa* bacterial cells immobilized in alginate. As shown in Figure 2a, both bacterial systems exhibited a similar trend, with current density increasing with the increase in OD_600_ from 0.1 to 0.5, followed by a decline at higher bacterial densities of 0.75 and 0.85. In the MEC based on alginate and *S. oneidensis,* the current density increased significantly, from 2.13 ± 0.15 A/m^2^ at OD 0.1 to a maximum of 4.08 ± 0.18 A/m^2^ at OD 0.5, representing nearly a 92% increase. However, further increases in OD resulted in a decline to 2.55 ± 0.10 A/m^2^ (OD 0.75) and 2.03 ± 0.12 A/m^2^ (OD 0.85). Similarly, the MEC based on alginate and *P. aeruginosa* showed an increase from 1.18 ± 0.13 A/m^2^ at OD 0.1 to 1.93 ± 0.18 A/m^2^ at OD 0.5 (63% increase), followed by a decrease at higher OD values (1.42 ± 0.06–1.45 ± 0.04 A/m^2^). This trend can be attributed to the balance of electroactive cell availability within the alginate matrix. A lower current density was observed at lower bacterial density, due to the limited electroactive cells available, while at the optimum OD 0.5, electroactive cells were available, resulting in higher current density. At a bacterial density higher than 0.5, the current density decreased, because a blockage was created when the electrode was fully loaded with the cells. The results indicated that OD_600_ 0.5 was the optimal bacterial loading for both systems. 

Previous studies demonstrated that the method of immobilization promotes electron shuttling, due to close contact between bacteria cells and the electrode surface, which generates current density by improving the attachment of cells to the electrode. In addition, they noted that the mediators produced by the bacterial cells are known to attach to the electrodes via their outer membrane, thus facilitating electron transport within the immobilized systems [16]. Gandu et al. reported that the immobilization of the exoelectrogenic biofilm of *G. sulfurreducens* on carbon cloth with alginate resulted in a current density of 11.52 A/m^2^ at 0.2 V, demonstrating the effectiveness of the immobilization strategy for enhancing electron transfer and electrode interaction. This study showed that hydrogel encapsulation improves bacterial retention and enhances electrochemical performance [1]. Similarly, Hirsch et al. demonstrated an alginate hydrogel bioanode system in which electroactive bacteria were immobilized within sodium alginate and applied in MECs, achieving a high current density of 4.14 A/m^2^ [17]. In contrast the present study investigates planktonic *S. oneidensis* and *P. aeruginosa* immobilized within alginate and systematically optimized bacterial density. The results indicated that an OD of 0.5 was optimal for bacterial density on both *S. oneidensis* and *P. aeruginosa* in immobilized anodes.

Electrochemical impedance spectroscopy (EIS) measurements yielded a Nyquist plot of electrodes incorporating bacteria, showing a clear change in the semicircle diameter with increasing bacterial loading (Figure 2b,c). The impedance data were fitted using R((Q)(R)(QR)(WR)). The semicircles correspond to the charge transfer. For *S. oneidensis* as shown in Figure 2b, the highest R_ct_, 23.8 Ω seen at OD 0.1, indicated poor initial electron transfer, attributed to low bacterial activity. The electrode prepared from OD 0.25 exhibited a semicircle corresponding to a lower R_ct_ of 17.2 Ω, which further declined to R_ct_ 7.3 Ω in the electrode prepared from OD 0.5. However, a higher density of bacteria was observed in the electrode with an OD of 0.75, resulting in an R_ct_ of 14.8 Ω, which was larger than that of the electrode with an OD of 0.5. The vertical low-frequency tail observed was ascribed to a larger increase in diffusion impedance, associated with an electrode with a more dense population of bacterial cells induced by the higher OD used in the preparation of these electrodes. The increase suggested excessive bacterial accumulation due to internal transport limitations. At OD 0.85, the semicircle became distorted, and the low-frequency tail became dominant, corresponding to an increased R_ct_ of 16.7 Ω, indicating a large bacterial accumulation and reduced ion mobility within the alginate matrix. The semicircle behavior confirmed that OD 0.5 provided the optimal bacterial activity. 

A similar trend was observed in the *P. aeruginosa*-based systems shown in Figure 2c. The semicircle diameter decreased with increasing bacterial loading up to OD 0.5, with R_ct_ values decreasing from 22.6 Ω to 17 Ω and reaching a minimum of 10.9 Ω. The smaller semicircle at OD 0.5 indicated improved electron transfer. At higher bacterial densities, OD 0.75 and 0.85, the semicircle broadened slightly [18], and the low-frequency diffusion region became more pronounced, with R_ct_ increased to 11.4 Ω and 15.2 Ω, respectively. Compared to *S. oneidensis*, the diffusion tail in *P. aeruginosa* was more prominent, likely due to the additional contribution of soluble redox mediator transport through the alginate matrix. These results indicated that changes in resistance were proportional to changes in bacterial density. The decrease in impedance was due to the cells’ viability, as bacteria adsorb to the electrode surface and their cell walls act as insulators. As bacteria grow and cell density increases, they form a blockage on the electrode surface, reducing the active surface area and increasing R_ct_. It is known that viable cells contribute to electrochemical activity, while cell death leads to an increase in impedance [19,20].

Overall, both LSV and EIS analyses indicated that OD_600_ 0.5 provided the optimal bacterial loading for alginate-immobilized *S. oneidensis* and *P. aeruginosa*. Therefore, this bacterial density was selected for further studies to investigate the effect of CB-nanoparticles on electrochemical performance. 

### 3.2. Effects of Carbon-Black Loading 

The role of CB-nanoparticles in the electrochemical performance of the immobilized bacterial systems was investigated by evaluating current density and EIS at an optimum bacterial density (OD_600_ = 0.5). CB-nanoparticle loadings of 1, 3, 5, 7, and 10 mg/anode were added to the alginate matrix. These experiments were conducted as a separate experimental set from the bacterial density optimization described in Section 3.1. MEC systems based on alginate-immobilized CB-nanoparticles were evaluated independently (in the absence of bacteria) to understand their contribution as conductive additives. They yielded relatively low current densities, ranging from 0.69 ± 0.12 to 1.12 ± 0.08 A/m^2^ as the CB-nanoparticle loading increased from 1 mg to 10 mg. The range 1–10 mg was selected to investigate the carbon loading and to avoid excessive aggregation and blockage at higher loading. Although an increase was observed with increasing CB loading, the overall current remained significantly lower than that of the bacterial systems. This behavior suggested that while CB improves electrode conductivity, it cannot independently generate significant current without bacteria; it thus can be considered an electrochemically inert component. 

**Figure 3:**
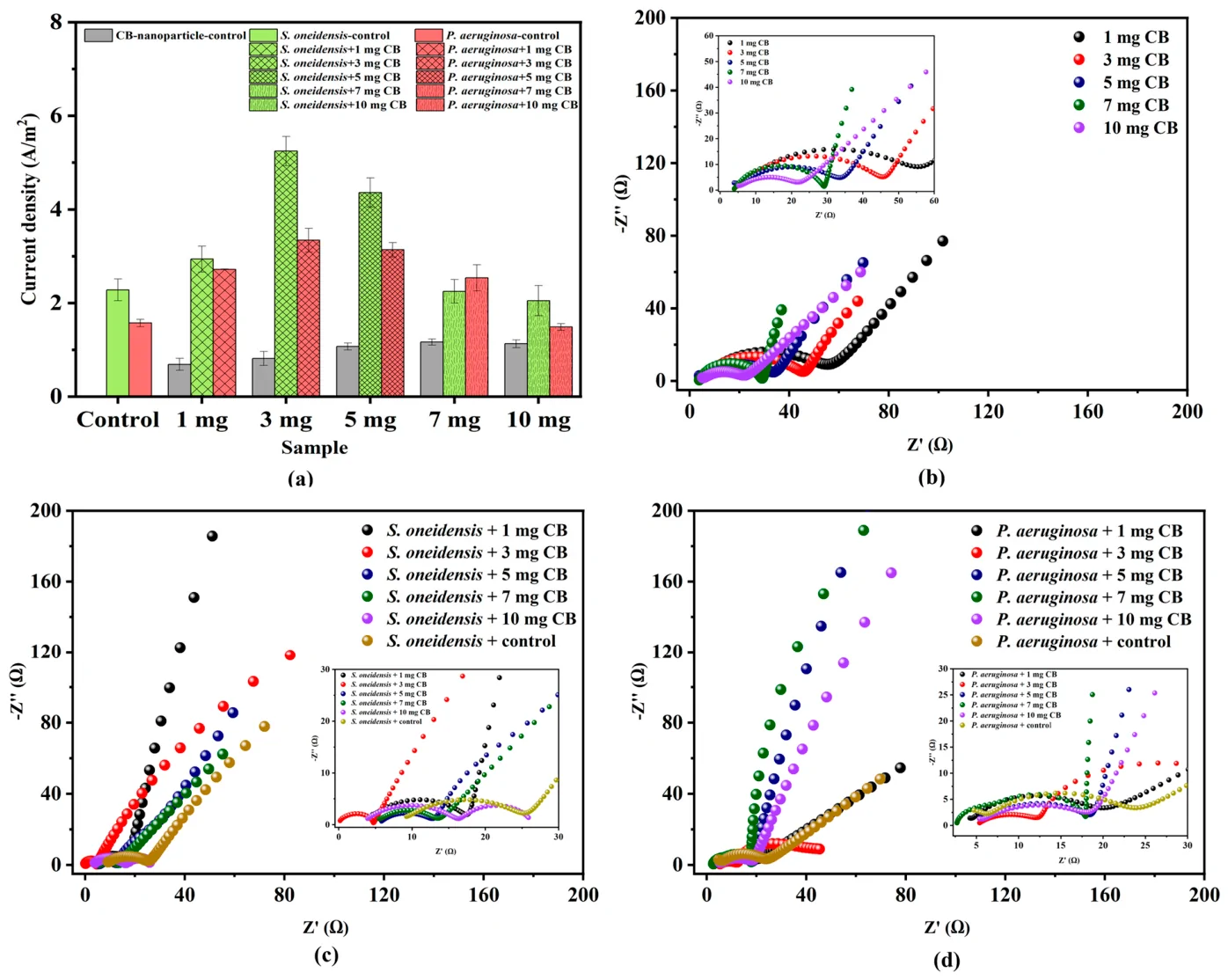
Alginate-immobilized *S. oneidensis* and *P. aeruginosa* in MEC systems with different CB-nanoparticle loadings from 1 to 10 mg: (**a**) current density at 0.8 V extracted from LSV measurements for both bacteria, (**b**) EIS of CB-nanoparticles, (**c**) EIS of *S. oneidensis,* and (**d**) EIS of *P. aeruginosa*.

A significant enhancement in electrochemical performance was observed when CB was combined with bacterial cells, demonstrating a strong synergistic effect. For MEC systems based on alginate-immobilized *S. oneidensis* (0.5 OD) with CB-nanoparticles, the current density increased from 2.28 ± 0.23 A/m^2^ (bacteria only) to 2.94 ± 0.27 and 5.24 ± 0.31 A/m^2^ at CB-nanoparticle loadings of 1 and 3 mg, respectively. However, at higher CB-nanoparticle loadings, the current density gradually decreased to 4.36 ± 0.31 A/m^2^ at 5 mg and then decreased to 2.25 ± 0.25 A/m^2^ at 7 mg and 2.05 ± 0.32 A/m^2^ at 10 mg. Similarly, for MEC systems based on alginate-immobilized *P. aeruginosa* with CB-nanoparticles (0.5 OD), the current density increased from 1.57 ± 0.07 A/m^2^ (bacteria only) to 2.72 ± 0.007 and 3.34 ± 0.25 A/m^2^ at 1 and 3 mg CB-nanoparticles, respectively. Then the current density decreased slightly to 3.14 ± 0.15 A/m^2^ at 5 mg, 2.54 ± 0.28 A/m^2^ at 7 mg, and 1.49 ± 0.07 A/m^2^ at 10 mg, as shown in Figure 3a and Table 1. 

The results suggested that CB-nanoparticles used for immobilization with bacteria in MECs enhanced bacterial attachment and electron transfer, leading to improved electrochemical performance. The maximum current density was obtained at a CB-nanoparticle loading of 3 mg for both bacterial systems. We concluded that at lower CB-nanoparticle loadings, the conductive network may be insufficient to facilitate electron transfer. In contrast, at higher CB-nanoparticle loadings, the aggregation and blockage may hinder ion diffusion and reduce effective cell–electrode interactions, leading to a decline in performance.

A comparison between the two MECs based on the bacterial anode revealed that *S. oneidensis* consistently exhibited higher current densities than *P. aeruginosa* under all tested conditions. At the optimal CB-nanoparticle loading of 3 mg, the *S. oneidensis* anode led to a current density of 5.24 ± 0.31 A/m^2^, significantly higher than the 3.34 ± 0.25 A/m^2^ observed for the *P. aeruginosa* anode. This difference can be attributed to the superior direct electron transfer capability of *S. oneidensis*, facilitated by outer-membrane redox-active proteins [21], whereas *P. aeruginosa* relies on mediated electron transfer mechanisms, which are comparatively less efficient under these conditions [22]. Previous studies by Cheng et al. modified a carbon cloth with a sodium alginate super-activated carbon composite hydrogel, which enhanced microbial attachment and electron transfer, resulting in a 4.6-fold increase in power density compared with a blank carbon-cloth anode [23]. Yang et al. developed a printed *S. oneidensis* bioanode using a sodium alginate-based bioink containing carbon black modified with riboflavin, resulting in a current density of 1.05 ± 0.05 A/m^2^ [24] Zhao et al. developed a PPy/EABs@Mag-CLF/SA capacitive hydrogel bioanode by immobilizing electroactive bacteria and magnetite-loaded carbonized particles within a polypyrrole-doped sodium alginate solution. The resulting bioanode enhanced extracellular electron transfer and achieved a power density of 71.88 W/m^3^ in a microbial fuel cell [25]. In comparison with these studies, the present study demonstrates that incorporating CB-nanoparticles in alginate containing planktonic electroactive bacteria significantly enhances current generation while enabling immediate electrochemical measurements without prolonged biofilm development. 

The EIS analysis further documented the current density, revealing clear impedance behavior across varying loadings of CB-nanoparticles. In the Nyquist plots, the semicircle region corresponded to charge transfer resistance (R_ct_). The low-frequency region represents a diffusion-control process. The CB systems without the bacteria showed high resistance as shown in Figure 3b, but there was a decreasing semicircle diameter observed with increasing CB loadings; the R_ct_ values decreased from 50.1 Ω CB at 1 mg to 16.9 Ω at 10 mg, as shown in Table 1, confirming that CB-nanoparticles enhanced the conductivity and facilitated electron transport within the system. For both *S. oneidensis* and *P. aeruginosa,* mixing with CB-nanoparticles altered the R_ct_ behavior compared with the bacteria-only control. The *S. oneidensis* control (without CB) exhibited higher R_ct_ and a border diffusion region, indicating slow electron transfer. Upon the addition of CB, the R_ct_ decreased. Thus, at 3 mg, the lowest R_ct_ value of 4.6 Ω was observed, which directly corresponded to the highest current density obtained for the *S. oneidensis*+CB 3 mg system, indicating that reduced charge transfer resistance resulted in more efficient EET. At higher CB loadings of 5–10 mg, the R_ct_ slightly increased compared with 3 mg as shown in Figure 3c. 

A similar trend was observed for the *P. aeruginosa* system, where *P. aeruginosa*+CB 3 mg exhibited the lowest R_ct_ value, 7.6 Ω, and the highest current density, 3.34 A/m^2^ (Figure 3d). The increase in R_ct_ at excessive CB loading suggested that increasing the CB-nanoparticle loading does not further enhance electron transfer performance. 

A correlation between current density and impedance clearly showed that enhanced electrochemical performance was strongly associated with reduced charge transfer resistance. These results highlight the importance of optimizing CB loading within the alginate matrix for the design of conductive polymer-based bioelectrodes.

Previous studies by Yuan et al. employed carbon-particle immobilization with bacterial cells; they were similarly reported to reduce diffusion resistance and improve electron transfer by enabling the electron transfer between the bacterial cells and the electrode surface [22]. The bacterial cells immobilized on the electrode surface exhibit a decrease in charge transfer resistance, ion transport resistance improves electron transfer, and diffusion limitations decrease as the mediators travel short distances. Similarly, the present study demonstrated conductive carbon-particle reduction in charge transfer resistance, with an increase in current density at optimal carbon-black loading. 

In conventional MEC studies, enhanced performance is typically associated with the formation of mature biofilms, which require extended incubation periods (typically weeks) to establish stable cell–electrode interactions. In contrast, the present study demonstrated that significant current densities could be achieved immediately after electrode preparation, without requiring biofilm development. This suggested that the alginate-facilitated immobilization strategy, combined with conductive pathways by CB-nanoparticles, enables immediate activation of the anode, thereby saving the time required to attain a fully grown and active biofilm. 

The observed increase in current densities and decrease in charge transfer resistance, particularly in the case of *S. oneidensis*, indicated that efficient electron transfer can occur even in the absence of a mature biofilm. This behavior contrasts with conventional systems, where biofilm formation is often a prerequisite for achieving high current outputs. The immediate electrochemical response observed in this study highlighted the effectiveness of an engineered electrode architecture in facilitating initial electron transfer processes.

### 3.3. Scanning Electron Microscope (SEM) Analysis 

**Figure 4:**
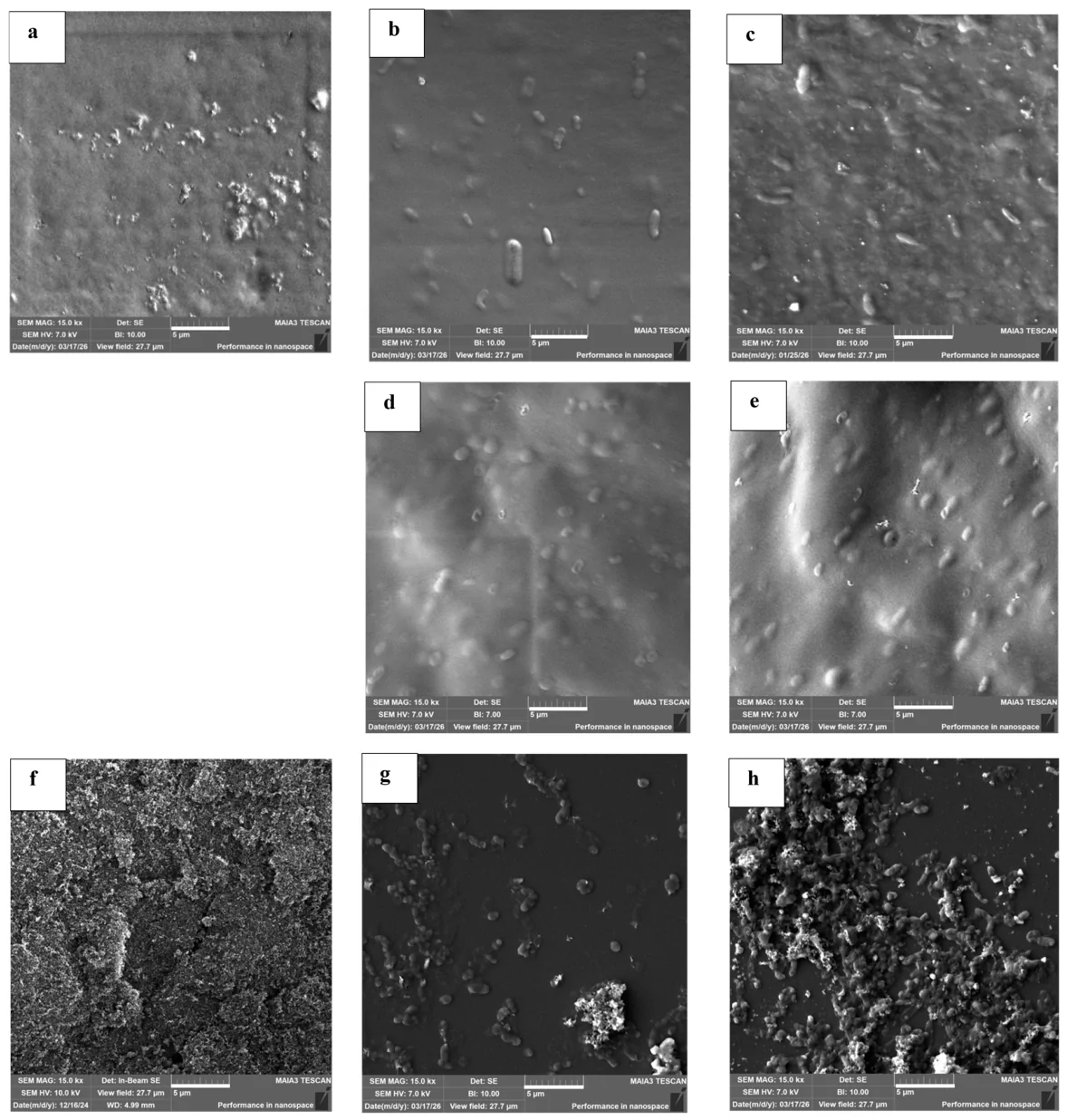
Alginate-immobilized anodes: (**a**) CB control, (**b**) *S. oneidensis* control, (**c**) *S. oneidensis*+CB, (**d**) *P. aeruginosa* control, (**e**) *P. aeruginosa*+CB. Samples without alginate: (**f**) CB-nanoparticle control, (**g**) *S. oneidensis*+CB-nanoparticles, (**h**) *P. aeruginosa*+CB-nanoparticles.

An SEM was employed to investigate the morphology and distribution of carbon black, bacterial cells, and their interactions under two conditions: (1) alginate-immobilized anodes and (2) CB-nanoparticle+bacteria samples prepared without alginate, and without an anode, to visualize the direct cell attachment. Figure 4a–e represent the alginate-immobilized samples. Figure 4a shows the alginate-immobilized, CB-nanoparticle-coated anode in the absence of bacteria. Figure 4b,c represent the *S. oneidensis* control (without CB-nanoparticles) and *S. oneidensis*+CB anodes. Similarly, Figure 4d,e display the *P. aeruginosa* control (without CB-nanoparticles) and *P. aeruginosa*+CB-nanoparticle anodes. In the alginate-immobilized samples, the bacterial cells and CB particles appeared embedded within the alginate, showing direct visualization of bacterial attachment to the carbon surface less distinctly. However, the SEM images confirmed the successful incorporation and uniform distribution of both bacterial cells and CB within the alginate on the anode surface. 

To further evaluate the direct interaction between the CB-nanoparticles and the bacterial cells, SEM analysis was performed on CB-nanoparticle+bacteria samples without the alginate immobilization, and without an anode (Figure 4f–h). The morphology of the CB (Figure 4f) revealed a porous and aggregated structure, providing a high surface area that is favorable for bacterial attachment. Figure 4g,h show *S. oneidensis* and *P. aeruginosa* with CB-nanoparticles. In both cases, bacterial cells were attached to the CB-nanoparticles, indicating strong physical interactions between the microorganisms and conductive material. This interaction between CB and bacterial cells suggested the formation of an electroactive bio-interface, which facilitates extracellular transfer by reducing the electron transport distance between the cells and the anode. Evidence of this interaction was supported by electrochemical performance which showed increased current density and reduced charge transfer resistance in the CB-nanoparticle-assisted systems.

### 3.4. Raman Spectroscopy 

Raman spectroscopy was performed on CB and bacteria–CB samples to characterize their spectral features and assess changes associated with the incorporation of CB into bacteria-containing samples. 

**Figure 5:**
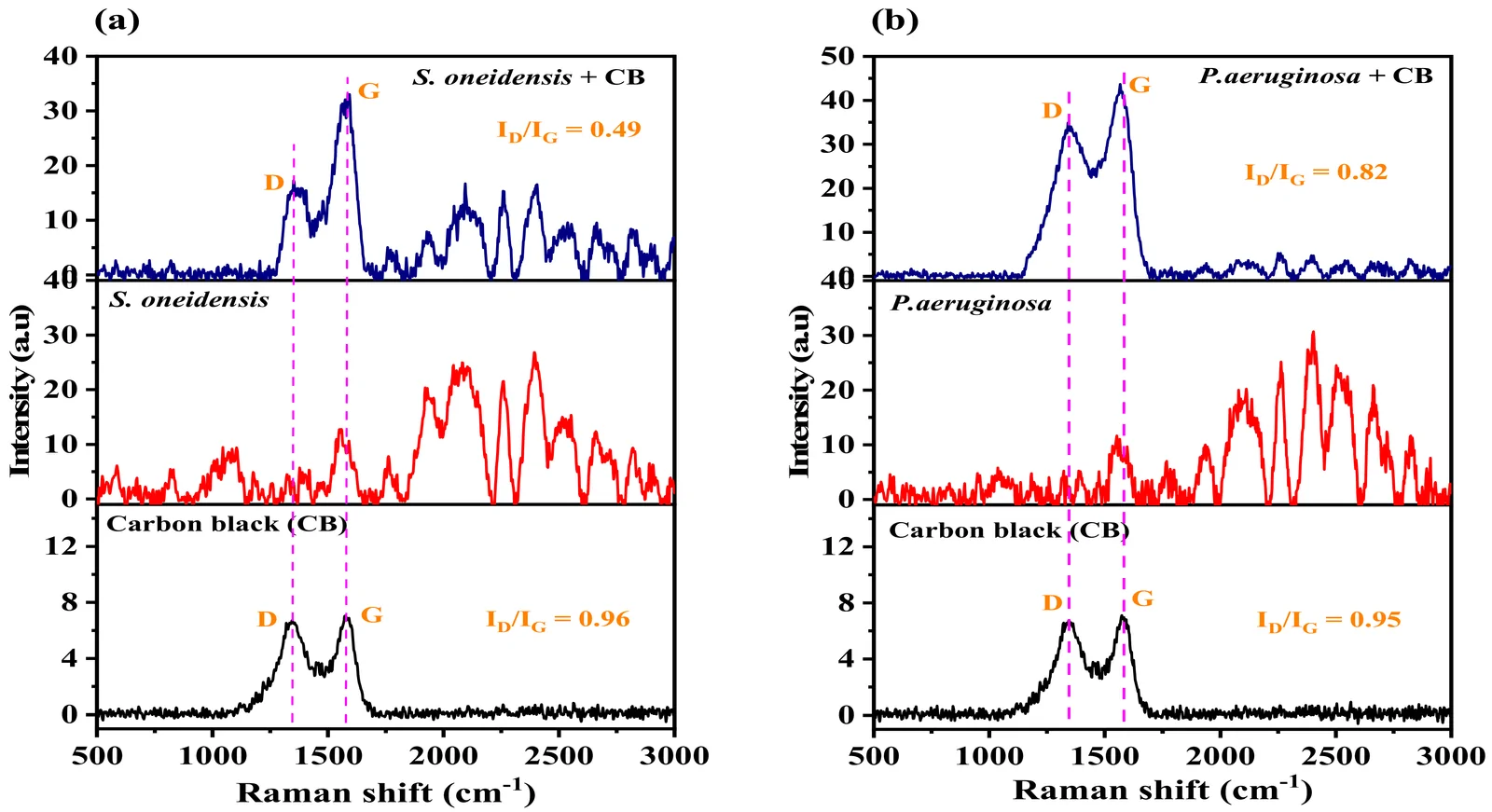
Raman spectroscopy of CB-nanoparticle, and bacteria-CB-nanoparticles samples: (**a**) *S. oneidensis* with CB-nanoparticles and (**b**) *P. aeruginosa* with CB-nanoparticles.

The Raman spectrum (Figure 5) of the CB exhibited two characteristic bands: the D band at 1343 cm^−1^ and the G band at 1583 cm^−1^. The D band is associated with the breathing mode of A1g symmetry arising from structural defects, while the G band corresponds to the in-plane vibration of sp^2^-hybridized carbon atoms (E2g phonon mode). The intensity ratio of these bands (I_D_/I_G_) is commonly used to evaluate the degree of disorder in carbon materials. For pristine CB, an I_D_/I_G_ ratio of 0.95 suggests a relatively disordered structure with a high density of defects. Meanwhile, for *P. aeruginosa*+CB-nanoparticles and *S. oneidensis*+CB-nanoparticles, the I_D_/I_G_ ratio further decreased to 0.82 and 0.49 respectively, suggesting Raman spectra of the bacteria-containing samples may include contributions from cellular components, which can overlap with the D and G spectral regions. Overall, together with the SEM and electrochemical results (LSV and EIS), the Raman spectra demonstrate spectral differences between CB and the bacteria–CB samples and support the presence of CB within the bacteria-containing samples. 

### 3.5. Proposed Electron Transfer Mechanism

**Figure 6:**
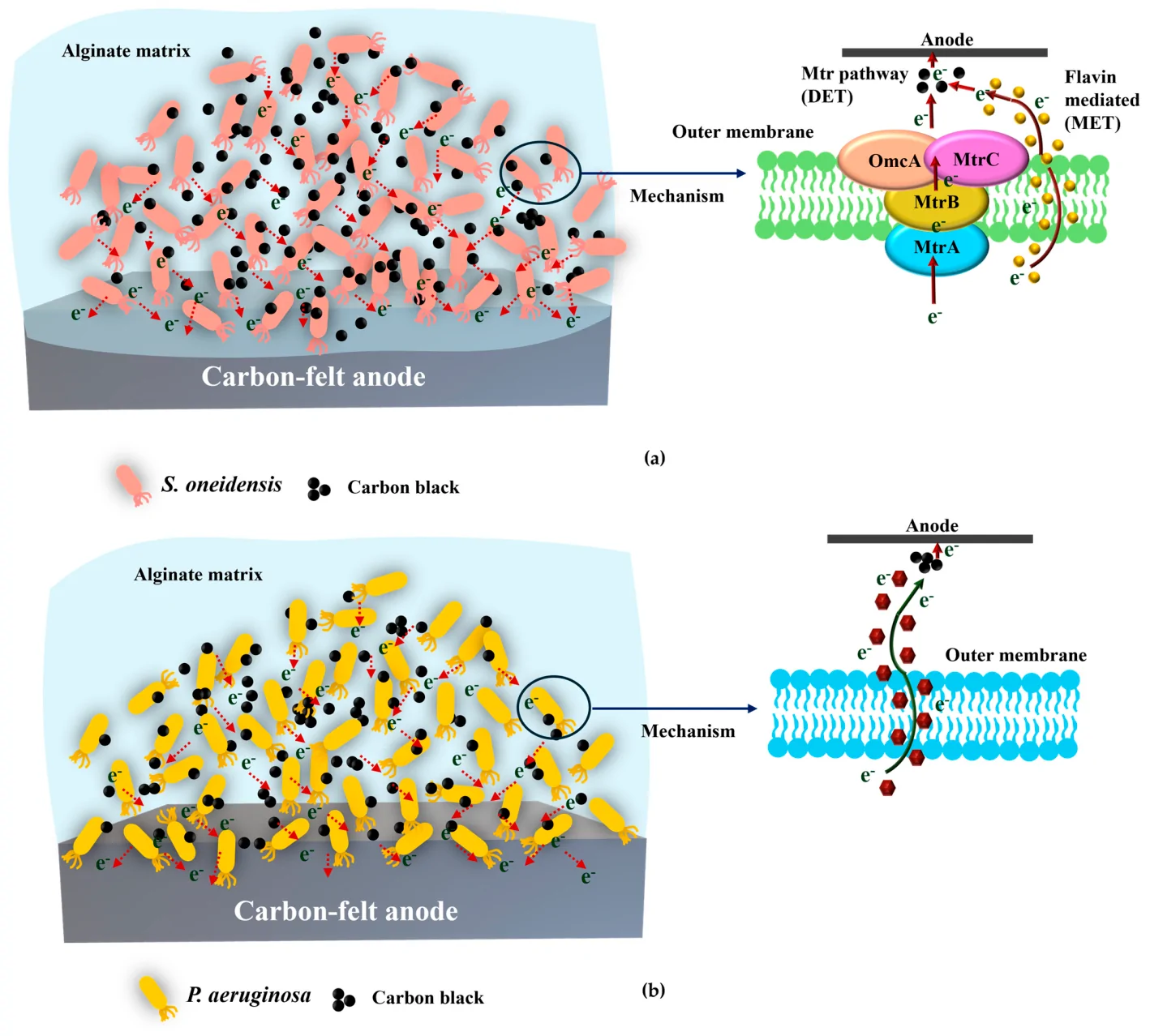
Proposed mechanism of (**a**) *S. oneidensis* and (**b**) *P. aeruginosa* interactions with CB-nanoparticles.

*S. oneidensis* and *P. aeruginosa* have distinctive EET mechanisms that exhibit physical appendages and soluble electron mediators. *S. oneidensis* (Figure 6) consists of C-type cytochrome complexes such as the MtrCAB/OmcA complex on its outer membrane. Electrons generated during substrate metabolism are transported through the Mtr pathway to outer-membrane cytochromes MtrC and OmcA, which can directly transfer electrons to the electrode through direct electron transfer (DET). In addition, *S. oneidensis* secretes flavins extracellularly, which mediate EET from MtrC/OmcA and electron shuttles diffusing between MtrC/OmcA and the electrodes, contributing to mediator electron transfer [4,21,26]. In contrast, *P. aeruginosa* relies on mediated electron transfer by producing redox-active metabolites known as phenazines, like pyocyanin [27]. These phenazines shuttle electrons from bacterial cells to the electrodes and increase the current density. The incorporation of CB into the alginate matrix is proposed to facilitate electron transfer by providing conductive networks within the alginate matrix. This interpretation is supported by the observed reduction in charge transfer resistance and increased current density through the SEM and Raman analyses, indicating interactions between the bacterial cells and carbon black. Therefore, the proposed mechanism remains an interpretation based on electrochemical and structural characterization. 

## 4. Conclusions 

This study investigated a bioanode configuration based on direct immobilization of planktonic electroactive bacteria within an alginate matrix coated onto a carbon-felt anode. The results demonstrated that bacterial density and CB-nanoparticle loading play critical roles in electrochemical performance. For both *S. oneidensis* and *P. aeruginosa,* an optimal bacterial density of OD_600_ 0.5 provided the highest current generation and reduced R_ct_; meanwhile, increasing bacterial density increased the R_ct_ and decreased current generation. Incorporation of CB-nanoparticles further enhanced electron transfer, with an optimum loading of 3 mg producing the higher current densities of 5.24 ± 0.31 A/m^2^ for *S. oneidensis* and 3.34 ± 0.25 A/m^2^ for *P. aeruginosa,* and also reduced R_ct_. SEM analysis demonstrated the association of bacterial cells with CB-nanoparticles, while Raman analysis revealed spectral differences between CB and the bacteria–CB samples. Overall, the alginate+CB immobilization strategy improved electrochemical characteristics and extracellular electron transfer for the prepared bioanodes. These results demonstrate the potential of alginate as a polymeric matrix for integrating immobilized electroactive bacteria with conductive CB-nanoparticles. This approach provides a basis for the further development of polymer-based bioelectrodes for bioelectrochemical systems.

## Figures and Tables

**Table 1:** Current density and EIS of alginate-immobilized bacteria with CB-nanoparticle immobilization on anodes.

	CB-Nanoparticle (mg/Anode)		*S. oneidensis*		*P. aeruginosa*	
	Current Density (A/m^2^)	R_ct_	Current Density (A/m^2^)	R_ct_	Current Density (A/m^2^)	R_ct_
Control			2.28 ± 0.23	16	1.57 ± 0.07	19.2
1 mg	0.69 ± 0.12	50.1	2.94 ± 0.27	13.1	2.72 ± 0.007	15.8
3 mg	0.81 ± 0.14	30.9	5.24 ± 0.31	4.6	3.34 ± 0.25	7.6
5 mg	1.07 ± 0.07	26.8	4.36 ± 0.31	7.2	3.14 ± 0.15	12.03
7 mg	1.16 ± 0.06	25.2	2.25 ± 0.25	8.2	2.54 ± 0.28	12.11
10 mg	1.13 ± 0.08	16.9	2.05 ± 0.32	11.94	1.49 ± 0.07	13.4

## Data Availability

The original contributions presented in this study are included in the article. Further inquiries can be directed to the corresponding author.
